# Structural Hierarchy of PA6 Macromolecules after Hydrostatic Extrusion

**DOI:** 10.3390/ma16093435

**Published:** 2023-04-28

**Authors:** Monika Skorupska, Mariusz Kulczyk, Piotr Denis, Dominik Grzęda, Anna Czajka, Joanna Ryszkowska

**Affiliations:** 1 Institute of High Pressure Physics, Polish Academy of Sciences (Unipress), Sokołowska 29, 01-142 Warsaw, Poland; mariusz@unipress.waw.pl; 2 Institute of Fundamental Technological Research, Polish Academy of Sciences, Pawinskiego 5B, 02-106 Warsaw, Poland; pdenis@ippt.pan.pl; 3 Faculty of Materials Science and Engineering, Warsaw University of Technology, Ul. Wołoska 141, 02-507 Warsaw, Poland; dominik.grzeda.dokt@pw.edu.pl (D.G.); anna.czajka2.dokt@pw.edu.pl (A.C.); joanna.ryszkowska@pw.edu.pl (J.R.)

**Keywords:** hydrostatic extrusion, polyamide 6, structure, thermal properties

## Abstract

This article presents the influence of severe plastic deformation by hydrostatic extrusion (HE) on the thermal and structural properties of polyamide 6 (PA6). During the hydrostatic extrusion process, a fibrous structure oriented along the extrusion direction is formed, which was visualized during microscopic observations. The degree of crystallinity was analyzed by differential scanning calorimetry (DSC). Wide-angle X-ray scattering diffraction (WAXS) analysis was used to partially characterize the PA6 structure after the HE process. The contents of various forms of the crystalline phase in PA6 samples before and after the HE process were analyzed in fragments of spectroscopy in infrared (FTIR). The favorable properties of PA6 after the HE process were obtained after deformation under conditions generating an adiabatic temperature higher than the glass transition temperature and lower than the temperature of the onset of melting of the crystalline phase. Thermal analysis using DSC allowed us to conclude that in the PA6 after the HE process generating deformations in the range of 0.68–1.56, the proportion of the crystalline phase α increases in PA6. As the deformation increases in the HE process, the crystalline phase proportion increases by 12% compared to the initial material (before HE). The glass transition temperature of PA6 is ca. 50.6 °C, reduced for the sample after the HE process at a small deformation of 0.68 (PA6_0.68) to ca. 44.2 °C. For other samples, T_g_ is ca. 53.2–53.5 °C. As a result of the analysis of WAXS diffractograms of PA6 samples after various deformations in the HE process, the presence of typical peaks of phases α_1_ and α_2_ and γ was observed. The results of the FTIR spectroscopic analysis confirm these observations that as the deformation increases, the proportion of the crystalline phase α increases.

## 1. Introduction

Polyamide 6 is a popular synthetic polymer widely used industrially and with many applications in engineering, including medicine. They are partially crystalline thermoplastics [1]. One of the methods of modifying PA6 to increase its strength properties is processing in the hydrostatic extrusion (HE) process. 

The effectiveness of this method in terms of the increase in the materials’ strength has been demonstrated to date for metals and metal alloys in many publications [2,3,4,5,6,7]. The HE processing of polymers was first described in 1964 [8]. One of the first papers showing the hydrostatic extrusion of Nylon 6 was published in 1978 [9]. The authors focused on the study of distortion grids and the influence of extrusion pressure on the polymer structure. Extensive data on hydrostatic extrusion parameters and properties of polymers after deformation processes were collected in the chapter “Polymers” of the book by N. Inoue [10]. The description of the HE process and the impact of its parameters on the strength properties of PA6 are presented in the paper by M. Skorupska et al. [11]. 

The hydrostatic extrusion process has been shown to increase strength properties such as tensile modulus, which rose by about 65%, and tensile strength, which rose by almost 500%. The changes in the tensile path observed are typical of changes caused by the stretching of polymers with increased pressure in the measuring chamber [12].

Based on the presented results, it can be concluded that the higher the strain applied during the HE process, the greater the tensile strength (σM) of the PA6 after the HE process. The tensile strength changes (σM) of the PA6 can be associated with changes in the structure of PA6 after the HE process, as indicated by the results of the studies presented by Li et al. [13]. 

The type of crystalline phase is most often described using wide-angle X-ray scattering (WAXS) [14,15,16,17,18,19,20,21]. In the form of α, the amide group and group (CH_2_)_5_ planes are parallel to each other, whereas in the form of γ, they are approximately perpendicular. Phase α is thermodynamically the most stable crystalline form [13]. The form γ can be converted into α by melting and then recrystallization, [22] by annealing at 160 °C in a saturated vapor atmosphere without any significant loss of orientation [23] and by applying stresses at room temperature. Intermediate crystalline forms occur between these phases [21,24,25,26,27,28,29,30,31,32,33,34,35].

Several methods are used to describe the degree of crystallinity of PA6—differential scanning calorimetry (DSC), density measurements, X-ray scattering, infrared and Raman spectroscopy, and nuclear magnetic resonance [36]. The determination of the degree of crystallinity allows us to understand the changes in the properties of PA6 after the thermomechanical processing process. Most often, DSC is used for this purpose. However, during the measurement in the annealing process, there is a rearrangement in the unstable crystalline phases, which causes the melting endotherm to contain thermal effects derived from the melting of the stable and unstable phases. Therefore, DSC research is complemented by other analysis techniques. Observation methods such as scanning electron microscopy (SEM) and polarized optical microscopy (POM) are also used to describe the structure.

To explain the reasons for the changes in the strength properties of PA6 after the HE process, the changes in the structure of these materials were analyzed. The results presented in the study by Skorupska et al. [11] indicate that the structure of PA6 changes after the HE process. Based on the SEM images presented in this study [11], it was found that during the HE process, a fibrous structure is formed with fibers arranged along the direction of extrusion in the HE process. To confirm these observations, microscopic observations were made using SEM and POM.

The impact of cold HE process parameters on changes in the structure of PA6 processed by this method was analyzed.

## 2. Materials and Methods

### 2.1. Material and Processing

The unmodified extruded polyamide 6 was purchased in the form of a rod with a diameter of 15.6 mm from MEGA-TECH s.c., Grodzisk Mazowiecki, Poland [37].

Samples of unmodified PA6 were subjected to the HE processes described in Table 1. Hydrostatic Extrusion was performed at a press designed and manufactured at the Institute of High Pressure Physics of the Polish Academy of Sciences (Warsaw, Poland). The press with a working chamber of 22 mm enables extrusion at pressures of up to 2 GPa. Prior to the HE processes, the rods were coated with a silicone-oil-based grease and squeezed out at a linear speed (v) in the range of 3.5–111 mm s^−1^ through forming a matrix with (die) the vertex angle 2α = 45° and diameters of 6, 7 and 8 mm.

The samples were subjected to true strain in the range of 0.68–1.90. The pressure values in the HE process were in the range of 100–430 MPa, which translated into the generation of heat in the material described by the adiabatic temperature in the range of 50–211 °C. All samples were extruded at the same extrusion rate of 5 mm s^−1^.

### 2.2. Experimental

#### 2.2.1. Tensile Test

The static tensile test (Zwick/Roell Z250, Ulm, Germany) was performed for samples with a diameter of 5 mm and a length of 10 mm at a tensile speed of 1 mm·min^−1^. 

#### 2.2.2. WAXS

WAXS experiments were carried out using a Bruker D8 (Bruker, Karlsruhe, Germany) Discover diffractometer operating at 1.6 kW nominal power with the Cu radiation source (wavelength 1.5418 Å). Data were recorded at room temperature, using a Bruker AXS Lynxeye 1-D detector (Bruker, Karlsruhe, Germany), in reflective mode. On the primary (source) side, a parallel Goebel-mirror optic was used, with a 1.2 mm linear slit and a 1 mm spot collimator. On the secondary (detector) side, a 2.5° divergence soller-slit was used.

#### 2.2.3. DSC

DSC analysis of PA6 was performed using a differential scanning calorimeter DSC Q1000 (TA Instruments, New Castle, DE, USA). Samples (ca. 7 mg) were closed in hermetic aluminum cups and heated and cooled at 10 °C min^−1^ in the temperature range 0 to 250. Based on the DSC thermograms obtained, melting peak distributions were performed using Origin Pro 8 and Fit Multi-peaks with Gaussian distribution. The crystalline weight fraction was computed from the enthalpy of the melting endotherm using the melting enthalpy of 100% crystallized ∆Hm 0(PA6) = 230 J/g [36] whatever the crystalline form.

#### 2.2.4. FTIR

PA6 samples were analyzed using absorption spectra obtained with a Nicolet 6700 spectrophotometer (Thermo Electron Corporation, Waltham, MA, USA) equipped with an attenuated total reflection (ATR) module. Each sample was scanned 64 times in the wavelength range of 4000–400 cm^−1^. The results were analyzed with the Omnic Spectra 8.2.0 software (Thermo Fisher Scientific Inc., Waltham, MA, USA). For each PA6 sample, a representation of three spectra was taken. The analysis of the spectra using OMNIC was carried out using the Gaussian distribution, assuming a linear baseline. Phase α and γ participation was calculated using the dependencies formulated by Dechant [38], for the crystalline variety α from Formula (1) and for the crystallographic variety γ:

(1) $$X_{IR\alpha }=\frac{A_{1030}}{A_{1075}}$$

(2) $$X_{IR\gamma }=\frac{A_{975}}{A_{1075}}$$

where: XIRα and XIRγ describe the proportion of polymorphic varieties of PA6 ordered regions, and A974, A1030 and A1073 are the fields of band absorbance at a wave number of 974 cm^−1^, 1030 cm^−1^ and 1075 cm^−1^, respectively.

#### 2.2.5. POM

Scrapings were cut from polyamide samples in the longitudinal orientation and crosswise to the direction of hydrostatic extrusion. The scrapings obtained by cutting were transferred to a basic microscope slide with a drop of immersion oil and covered with a flat cover slip. The cutting of the scrapings was performed with the use of a Leica EM FC6 ultramicrotome (Leica Microsystems, Wetzlar, Germany), using the following cutting parameters: feed speed—0.40 mm s^−1^, slice thickness—2400 nm, room temperature. The slices were cut with glass knives. Observation of the samples was carried out using a Biolar P1 polarization–interference microscope (PZO, Warszawa, Poland), using a 20X objective and crossed polaroids, also at room temperature. Observations were carried out in dark and bright fields. The parameter of spherulites and fibers present in a polarized optical microscopy (POM) image was quantified using ImageJ (Fiji ImageJ 2.9.0). 

#### 2.2.6. SEM

Using a ZEISS SEM microscope (ZEISS, Jena, Germany), model ULTRA PLUS with a Gemini column (Jena, Germany), structural analysis was performed. Samples were frozen in liquid nitrogen for 5 min and then fractured by bending. The surface of these cracks was observed. The samples were dusted with a layer of gold before observation.

## 3. Results and Discussion

The HE process of polyamide 6 was carried out at different sample deformations in the range of 0.68–1.90 resulting in an extrusion pressure in the range of 100–450 MPa. Hydrostatic extrusion was carried out at the same linear deformation rate of 5 mm min^−1^. The HE process at 430 MPa was an unstable process, resulting in changes in the strength properties of PA6_1.90 samples not being analyzed.

### 3.1. Mechanical Properties of PA6 after Cold HE

**Figure 1:**
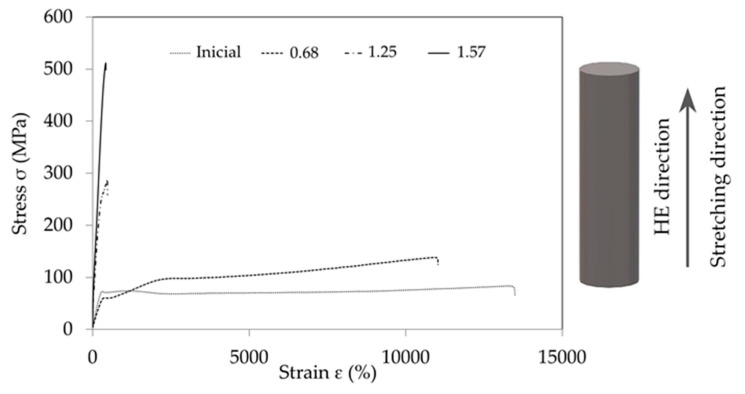
Examples of tensile curves before hydrostatic extrusion (Inicial) and after the process at different true strains (ε = 0.68, ε = 1.25, ε = 1.57).

As a result of the research on strength properties, some of which are presented in the study by Skorupska et al. [11], it was found that during the stretching of the example samples subjected to the HE process, the nature of the tensile curves changed (Figure 1).

The tensile PA6 samples are described by the tensile curve typical of materials in the viscous elastic state, as is the PA6_0.68 sample. In contrast, samples PA6_1.25 and PA6_1.56 are described by tensile curves typical of materials in a brittle elastic state, also called vitreous. 

The observed changes in the tensile curves are typical of changes caused by the stretching of polymers at increased pressure in the measuring chamber [12]. Based on the results presented in Figure 1, it can be concluded that the higher the deformation in the HE process, the greater the modulus of elasticity of PA6 after the HE process. Changes in the modulus of elasticity of PA6 can be associated with changes in the morphology of PA6 after the HE process, as indicated by the results of the research presented by Li et al. [13]. Based on the research presented in this study, it was found that the modulus of elasticity of PA6 in the form of α is more than twice as high as for PA6 in the form γ.

### 3.2. Structural Investigations

**Figure 2:**
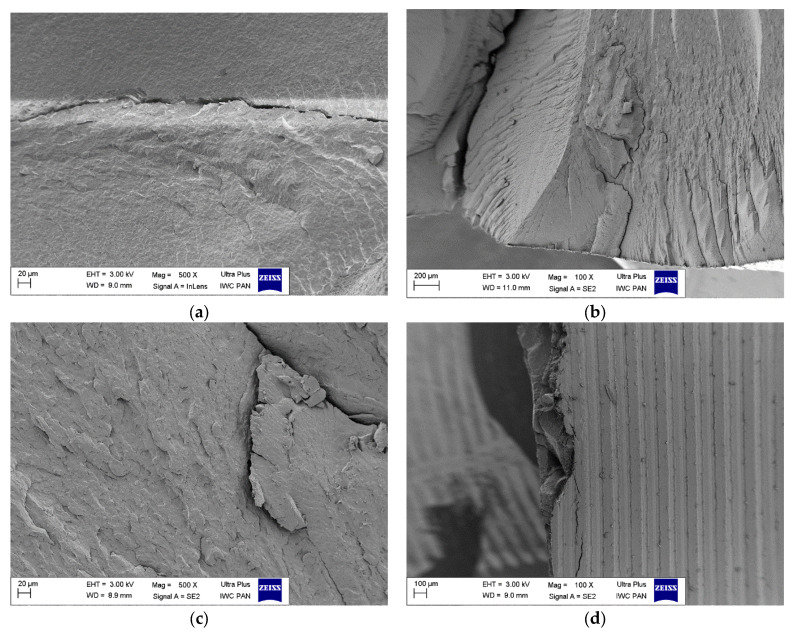

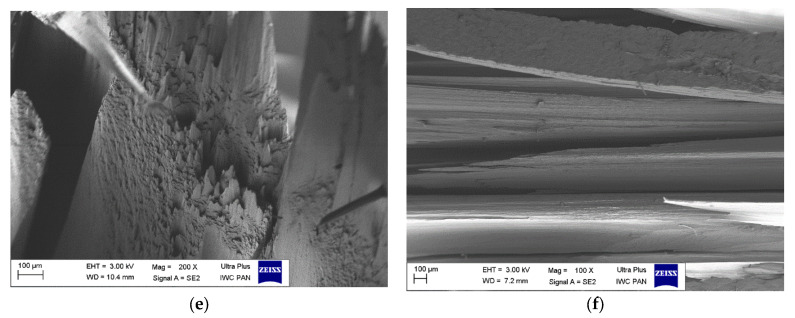
Scanning electron microscopy (SEM) images of cryo-fractured transverse cross-sections of the polyamide PA6 in initial state: PA6_across (**a**), PA6_along (**b**). Rod after cold hydrostatic extrusion HE with true strain ε = 0.68: PA6_0.68_ across (**c**), PA6_0.68_along (**d**) and rod after cold HE with ε = 1.57: PA6_1.56_across (**e**), PA6_1.56_along (**f**).

After the hydrostatic extrusion process, polyamide PA6 crystallizes. During crystallization, spherulites are formed from the lamellae [13]. Images of the sample fracture structure made across the extrusion direction (PA6 across) and along the HE direction (PA6 along) are shown in Figure 2.

The sizes of these ovals are less than 20 μm. It follows from the results presented by Osorio et al. [39] that the size of spherulites in the PA6 samples they studied varied in the range of 10–20 μm.

The PA6_0.68_across fracture is different from the PA6_across fracture. The elements of the surface structure of this fracture have the character of oval islands with elongated shapes. On the other hand, at the fracture of this sample along the HE direction (PA6_0.68_along), elements in the form of bands arranged parallel to each other are visible, these bands differ in width, and the narrower bands have a width of approx. 50 μm, and the wider ones of ca. 80 μm. They are separated by a band with a width of 40–60 μm. It can be assumed that these bands were formed by straightened-out fragments of lamellae forming spherulitic structures from different varieties of the PA6 crystal phase (α and γ). The structure of the PA6_1.56 sample fractures is different. The band structures formed in this material are characterized by such high strength that it was impossible to perform a flat fracture in the across direction to the HE direction. The PA6_1.56_across sample fractures allow broken and pulled fiber bundles to be observed. At the fracture formed along the HE direction (PA6_1.56_across), wide fiber bundles of 140–180 μm are visible, as well as narrow bundles with a much smaller size of ca. 60–80 μm, which are separated by gaps of approx. 60 μm. As in the case of the PA6_0.68_along material, the band structures were formed in this material. They are formed from straightened fragments of spherulite lamellae of different varieties of the PA6 crystalline phase (α and γ). Higher pressure during the HE process at deformation ε = 1.56 caused more agglomerates of bands connected by hydrogen bonds to form than in the material under deformation ε = 0.68. 

Analysis of sample fracture images indicates that an increase in true strain during the HE process results in an increase in the cross-sectional diameter of the fiber bundles formed during the HE process. This change in the structure of the materials is probably the reason for the significant increase in the strength of PA6 after the HE process.

**Figure 3:**
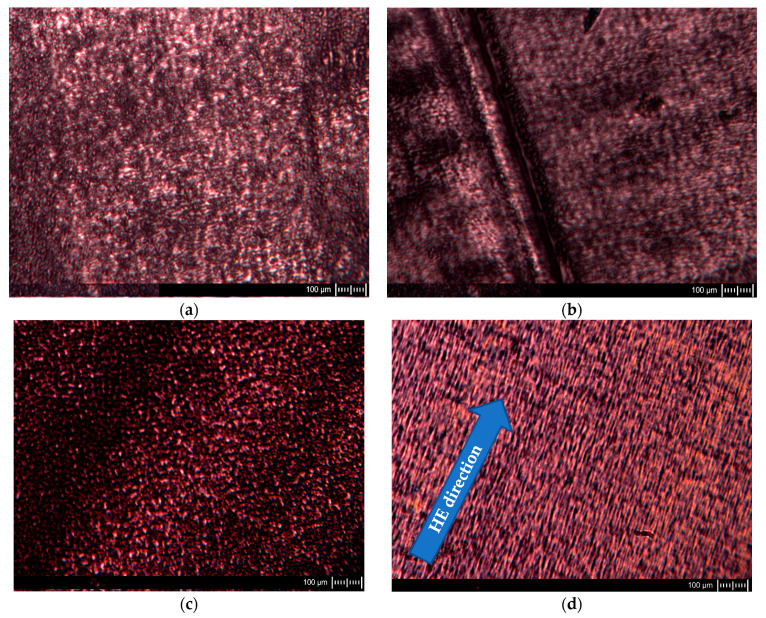
Example images of POM sample cuttings before (**a**,**b**) and after HE process (**c**,**d**). The samples labeled across: (**a**,**c**) were made across the extrusion direction for PA6 and across the HE direction for samples after this process, and the samples labeled (**b**,**d**) along the extrusion direction for PA6 and along the HE direction for samples after this process.

Observations of ultramicrotome cuttings were also made with a polarized scanning transmission electron microscope; exemplary images are shown in Figure 3.

**Figure 4:**
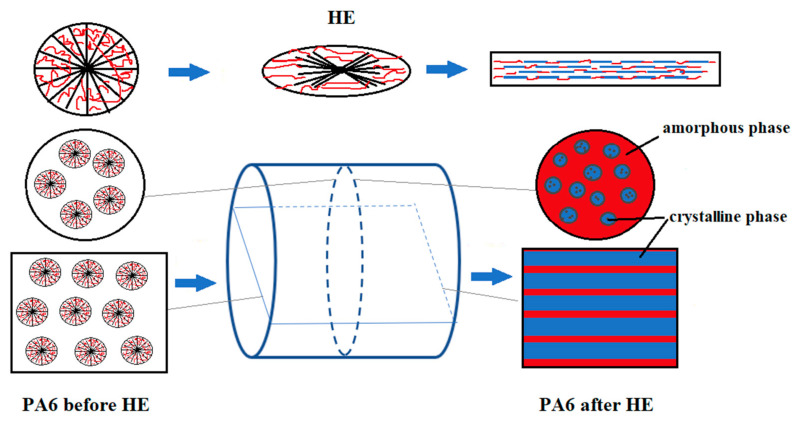
Diagram of changes in the structure of PA6 sample fractures before and after the HE process.

The images of the PA6 sample cuttings at baseline observed in POM show oval-shaped structural elements reflecting the shape of the spherulites formed in these samples. A similar pattern was observed for the sample in the case of a cut made across the extrusion direction as well as along the extrusion direction. The images of PA6_1.56_across sample cuts made across the HE direction are similar to the PA6 sample cutting images. Images of the structure of the sample PA6_1.56_along cut made along the HE direction differ significantly from that made across the HE direction. The structural elements of the cuts made along the HE direction have the shape of rods arranged in a regular manner parallel to each other. Based on the SEM and POM images, a scheme of changes in the structure of PA6 caused by deformation in the HE process is proposed, which is presented in the diagram shown in Figure 4.

During the HE process, the deformation of partially crystalline polymers in the solid state occurs. This process is possible at a temperature above the glass transition temperature (T_g_) and below the melting point (T_m_). During deformation in this temperature range, cavitation phenomena [40] and phenomena resulting from crystal plasticity can occur, causing crystallite regrouping [41,42]. In the HE process, the optimal parameters of the extrusion process are achieved below T_m_, and, therefore, it can be assumed that the course of the deformation process depends on the degree of crystallization of the processed material since crystalline areas limit the movement of molecules [43] and may also be the reason for the strengthening of materials due to the orientation of macromolecules [44].

### 3.3. Results of Diffraction Analysis

**Figure 5:**
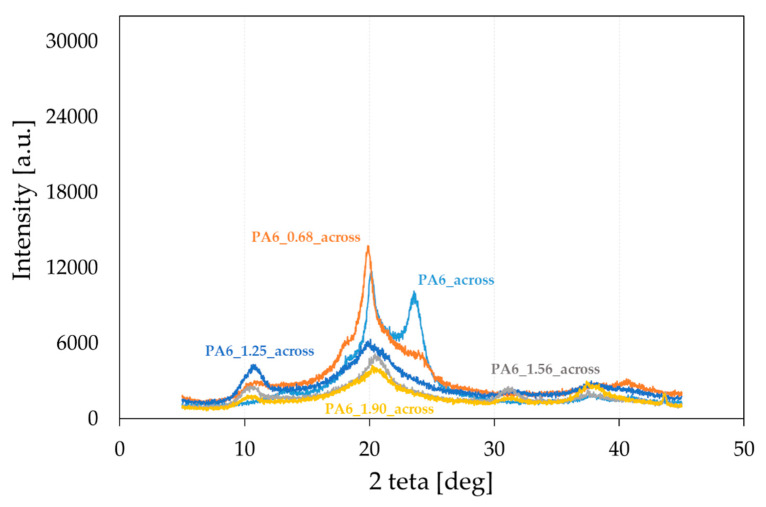
Diffractograms of PA6 samples before the HE process (PA6) and after the HE process at variable true strain. Diffractogram obtained for a cross-sectional sample.

**Figure 6:**
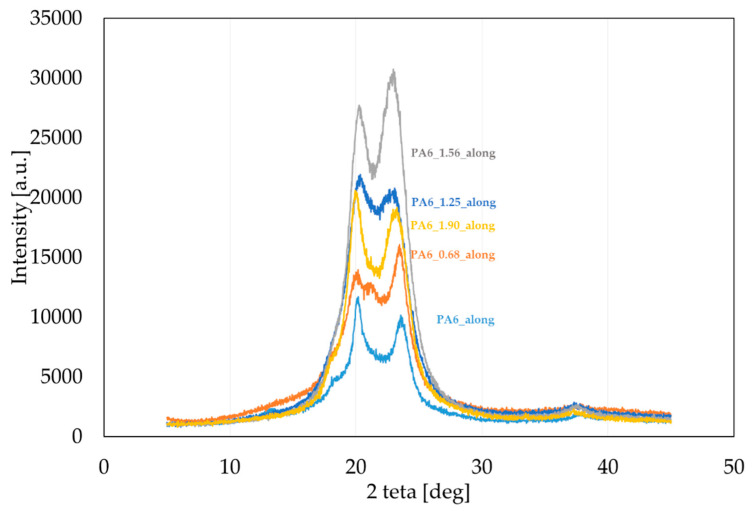
Diffractograms of PA6 samples before the HE process (PA6) and after the HE process at variable true strain. Diffractogram obtained for a sample from a cross-section made along the HE direction.

PA6 occurs in two thermodynamically equilibrium crystalline forms α and γ, and one mesomorphic form β [31,45,46]. During slow cooling, the molten state forms phase α, and during rapid melting, phase γ. The formation of the γ phase was also observed in nanocomposites, its nucleation was caused by nanofillants such as clay, zinc oxide and mica [47,48,49,50]. In order to characterize the structure of PA6 after the HE process, WAXS diffractograms were performed for selected PA6s before and after the HE process. The cross-sectional surfaces of the samples (across) (Figure 5) and the cross-sectional surfaces made along the extrusion direction (along) (Figure 6) were analyzed. Based on the analysis of the diffractograms, listed in Table 2 are reflections seen in the diffractograms shown in Figure 5, obtained after the HE process at different degrees of deformation. 

At room temperature, there can be two phases, α and γ in PA6. According to [16,23,51], the characteristic peaks of the α_1_-phase appear at an angle 2 theta between 20.2 and 20.6° (200, α_1_) and the α_2_-phase at an angle 2 theta = 24° (002 + 202, α_2_). However, for the γ-phase, there are reflections coming from two forms of this phase: at an angle of 21° (100, γ_1_) and 22.5°(201 + 200, γ_2_) [52]. 

Based on the introduction to the publication [53] and the information contained therein, based largely on the long and intensively studied structure of PA6 in the history of polymer research, it is possible to briefly characterize the phases α and γ:

- α is composed of PA6 extended chains while γ form is composed of pleated chains;
- Mainly intra-sheet hydrogen bonding is present in α form, while in the γ form inter-sheet hydrogen bonds are dominant;
- Young modulus of α form is considered to be higher than γ form (due to differences in ordering of the structures in both forms);
- α form is considered to be more thermodynamically stable.

According to the above characteristics, it can be considered that the main differences between the α and γ forms are the shape of the chains (extended vs. pleated) and the hydrogen bonding direction which results in different crystal structures and d-spacings (hydrogen bondings in pleated γ chains are tilted by about 60° compared to the bonding direction of the α form). We consider that it is relatively safe to assume that mechanical deformations which accompany the extruding process may result in changes in the shape of the chains (it may pleat them or extend them) as well as in the directions of chains.

It was found by analyzing the diffractograms PA6_across and PA6_along that the reflections coming from phase α_1_ and α_2_ occur at 2 theta = 20.01° and 23.49° and 2 theta = 20.09° and 23.65°, respectively. The reflection from phase γ occurs at 2 theta = 21.21° and 2 theta = 20.56°, respectively. The peak intensities for the output PA6_across and PA6_along are similar, suggesting that the samples in the initial state are close to isotropic structures. Diffractograms of samples after the HE process made across (Figure 5) and along the HE direction (Figure 6) differ markedly in the intensity of phase-related reflections α_1_ and α_2_ and γ, which indicates their anisotropic structure.

In the diffractograms of HE-deformed samples taken across the direction of deformation (Figure 5), reflections were observed from phase α_1_ at 2 theta = 19.80–20.26°, and this reflection does not occur for the sample PA6_1.56_across only. For the sample PA6_0. 68_across, there is also a reflection from phase α_2_ at 2 theta = 24.07°. Reflections from phase γ occur in the range of 20.45–21.06°; the greater the deformation in the HE process, the closer the reflection position is to 20.4°. On the other hand, in diffractograms of the surface of samples after the HE process made along the direction of extrusion (Figure 6), there are peaks coming from phase α_1_ at 2 theta = 19.89–20.20° and from phase α_2_ at 2 theta = 23.05–23.49°. The reflection from phase α_2_ moves towards lower angles at deformations 0.68, 1.25 and 1.56. For phase γ reflections occurring in the range of 20.99–21.88°, and for samples under deformation 0.68, 1.25 and 1.56, the reflections from this phase move towards angles closer to 22°. 

A summary of the results of the analysis of reflections in the obtained diffractograms is presented in Table 2. 

**Figure 7:**
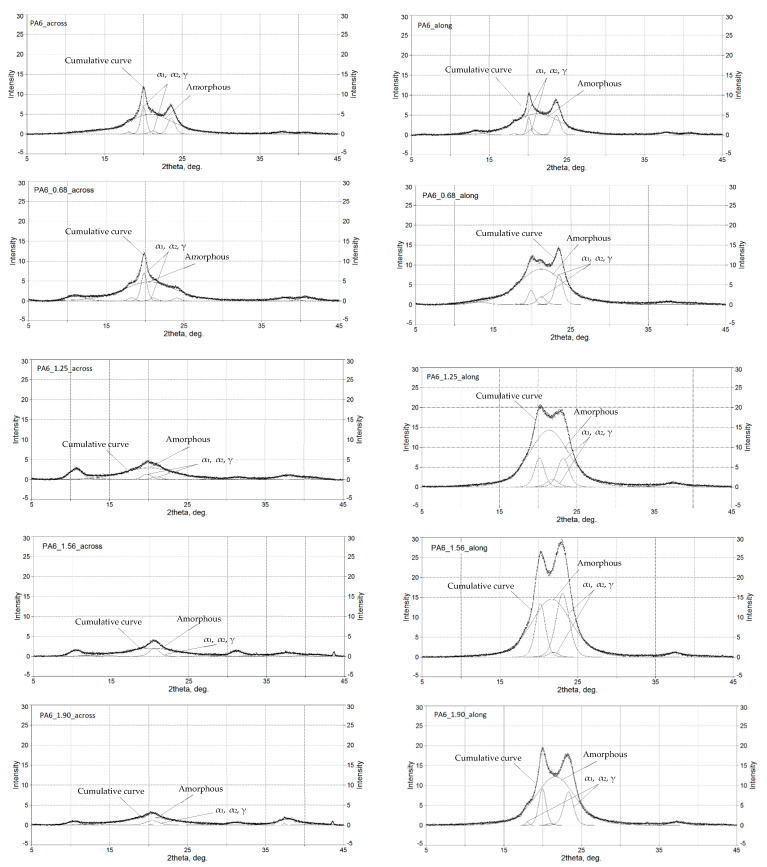
Distribution curves of diffractograms of the tested materials.

The deconvolution of the WAXS spectra is shown in Figure 7. The results of the analysis of the deconvolution into component bands are shown in Table 3. 

In the longitudinal direction (along the deformation direction):

– For deformations 0.68, 1.25 and 1.56, an increase in the contribution of the alpha phase;
– Components are observed, and therefore an increase in the proportion of longitudinally directed chains (at the same time, a slight increase in the gamma phase component to deformation of 1.25 and then its sharp decline. In quantitative terms, however, the contribution of the gamma phase to the structure is small relative to the alpha phase in this direction, a maximum of about 12% for the 1.25 sample, and only about 3% of the contribution taking into account the amorphous phase);
– The high contribution of the alpha structural component can therefore be reflected in the high rigidity of the material, increasing with deformation up to a value of 1.56 deformation. It can be seen that for the maximum deformation 1.90, the areas of both the alpha and gamma component peaks decrease, so perhaps the material reaches its maximum orientation and the resulting strengthening between 1.56 and 1.90, while at deformation 1.90, the structure undergoes degradation, e.g., chain breakage, or other deformation if we assume that this is possible, or reorientation, deviations from this direction.

In the across direction:

– With an increase in deformation, the contribution of alpha components decreases, which seems logical because we deform the structure in the perpendicular direction and in the transverse direction, possibly occurring longitudinal chains of the alpha structure are deflected and deformed;
– For deformations 0.68, 1.25 and 1.56, the contribution of the gamma component increases systematically (reaching 100% of the contribution for deformation 1.56—there are no alpha components here);
– For deformation 1.90, we have the alpha component.1 but note that its surface area is absolutely marginal (0.06 is the level of 2% in the crystalline components and below 0.5% in the entire structure of the material taking into account the amorphous phase);
– What is more interesting is that for the 1.90 deformation, just as it was in the longitudinal direction for the alpha component, the surface of the gamma component also decreases, so the conclusion is similar to the above: the 1.90 deformation escapes from some of the systematics observed for the 0.68–1.56 range, and the structure changes in a less controlled and predictable way.

The HE process results in a clear increase in phase α participation to PA6. In the study by Peppin [21], it has been shown that in PA6, there is a transition γ→α caused by mechanical impacts regardless of the temperature at which the deformation process took place, and Murthy [54], in the process of studying the PA6 γ fibers uniaxially drawn, observed transition from phase γ to a metastable structure.

### 3.4. Spectroscope Analysis Results

**Figure 8:**
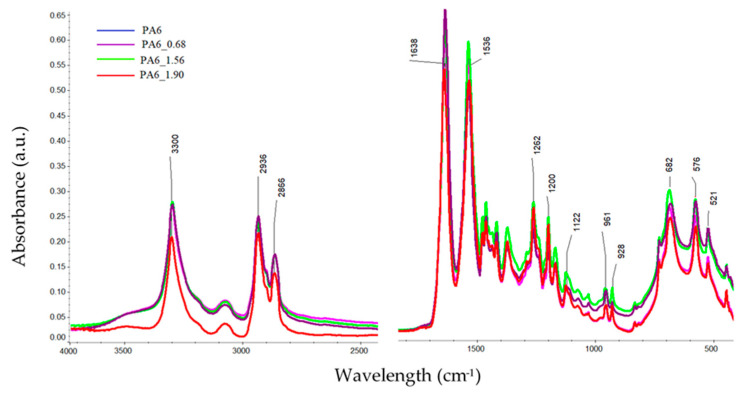
FTIR spectra of PA6 before and after the HE process at variable deformation.

The FTIR spectra of the tested PA6 before and after HE was analyzed, with example spectra, are shown in Figure 8. There were no significant shifts observed in the positions of the bands in the spectra of the samples, but there was a change in their intensity. Differences in the band intensities are also noted for the bands associated with the crystalline phase of the variety α: 1200, cm^−1^, 1029, 960 and 929 cm^−1^ [38].

**Figure 9:**
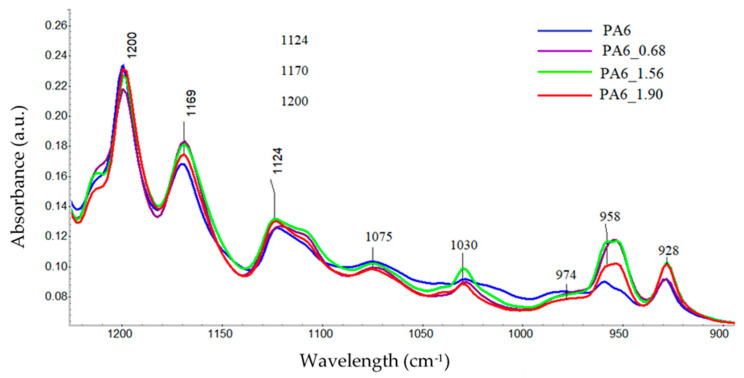
The spectral range typical of the crystalline phase in PA6 before and after the HE process.

To analyze the content of different forms of crystalline phases in PA6 samples before and after the HE process, fragments of spectra were analyzed for ranges typical of the PA6 crystal phase, i.e., 1200–900 cm^−1^ [38]. The comparison was made for spectra collected from the center of cross-sections of samples made along the HE direction (Figure 9).

**Figure 10:**
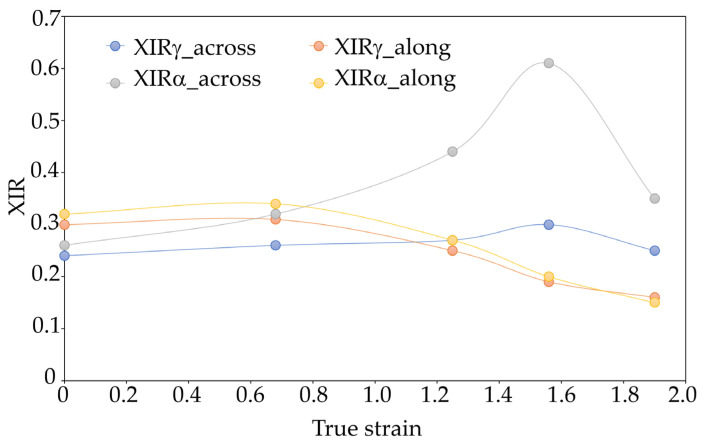
Crystallinity indices of various varieties of crystallographic PA6 samples before and after the HE process at different deformations determined on the basis of FTIR. Results of the FTIR analysis of the central part of the cross-section of the samples (across) and the cross-section made along the HE direction (along).

Crystallinity indices of different varieties of crystallographic PA6 samples before and after the HE process at different deformations determined on the basis of FTIR are presented in Figure 10. 

The nature of the changes in the crystallinity index of individual crystalline varieties α and γ determined on the basis of the FTIR analysis confirms the observations from the WAXS analysis that in the case of the PA6_1.56 sample, in the cross-section made along the HE direction, most of the crystalline phase takes the form of α. This result indicates a much higher orientation of the structural elements and a higher degree of ordering of the PA6_1.56 sample compared to other materials.

### 3.5. Results of Thermal Analysis Using Differential Scanning Calorimetry

**Figure 11:**
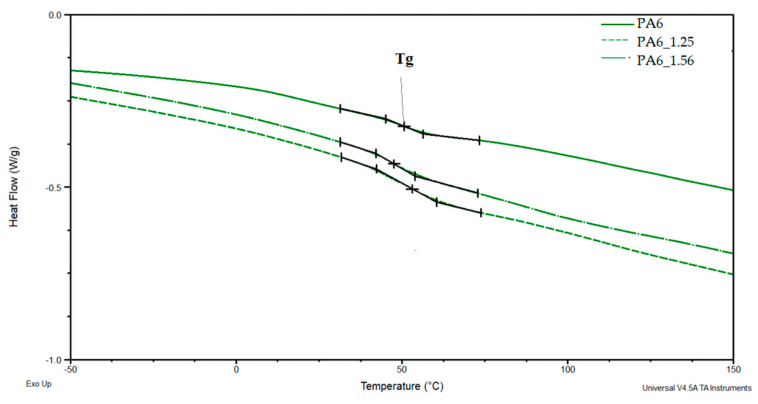
DSC PA6 thermograms before and after the HE process at different deformations in the range of glass transition temperature—T_g_.

**Figure 12:**
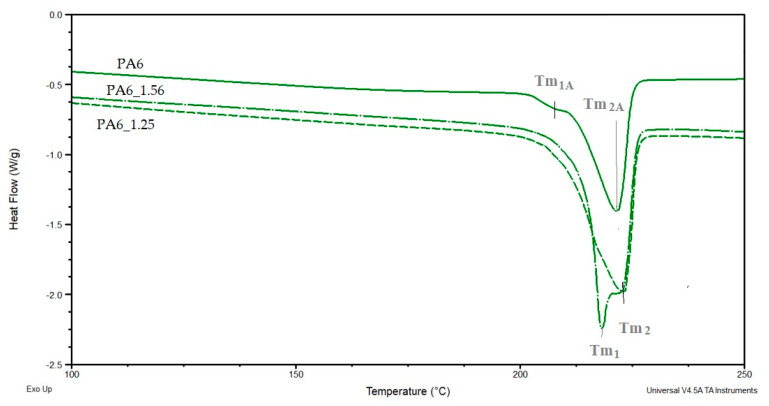
DSC PA6 thermograms before and after the HE process at different deformations in the melting point range—T_m_.

Figure 11 and Figure 12 show a comparison of selected DSC PA6 thermograms before and after the HE process at different deformations, taken from the central part of the rod.

**Figure 13:**
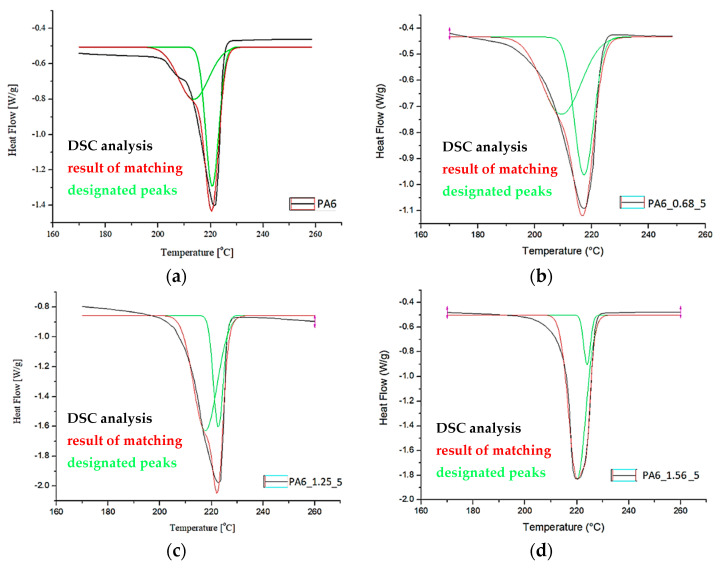

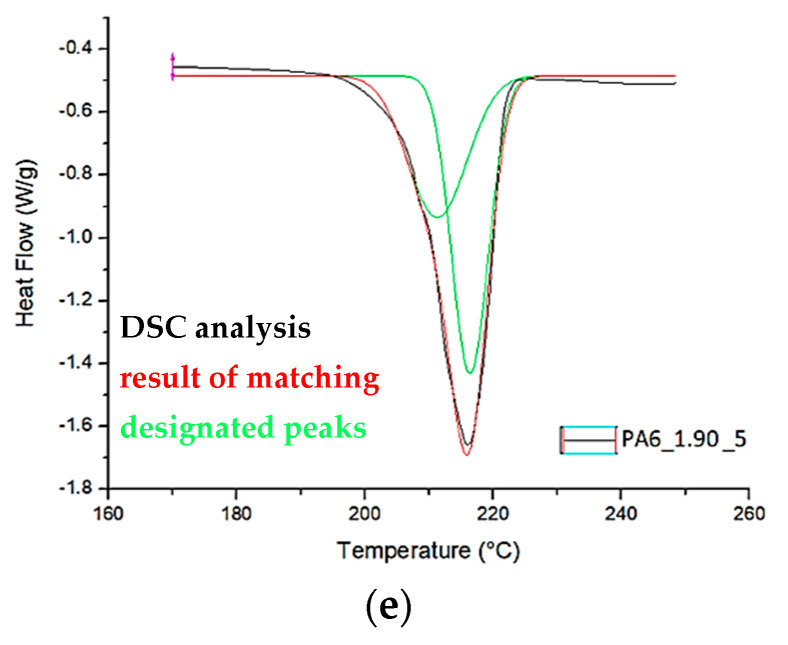
Distribution of the multiplet band from the melting of PA6 crystalline phase before (**a**) and after the HE process (**b**–**e**).

Based on the DSC thermograms (Figure 11 and Figure 12), the temperatures of glass transition T_gA_ and crystalline phase melting (T_m1_ and T_m2_) of the PA6 were determined before and after the HE process leading to different sample deformations. The results of the distribution of the multiplet peaks associated with the crystalline phase melting are summarized in Figure 13. 

The melting enthalpy was also determined for each of the peaks that make up the multiplet peak associated with the crystalline phase melting. The results of the DSC analysis and the distribution of the crystal phase melting-related multiplet peaks are summarized in Table 4.

The glass transition temperature of PA6 is ca. 50.6 °C, reduced for the sample after the HE process at a small deformation of 0.68 (PA6_0.68) to ca. 44.2 °C. For other samples, T_g_ is ca. 53.2–53.5 °C. Changes in the microstructure of PA6 samples after the HE process cause slight differences in the mobility of the macromolecules of this polymer. 

A multiplet endothermic peak with a melting point of the crystalline phase was observed in the PA6 sample at T_m1_ = ca. 214 °C and T_m2_ = ca. 221 °C. The first temperature can be associated with the melting of the crystalline phase of the variety γ which is usually ca. 214 °C [39], the second temperature T_m2_ for PA6 according to Millot et al. [15] is associated with phase α.

In samples PA6_0.68 and PA6_1. 90, temperatures T_m1_ and T_m2_ are ca. 210 °C and ca. 217 °C. Both temperatures are lower than the melting points associated with the melting of phases γ and α. This indicates that these phases are defective.

**Figure 14:**
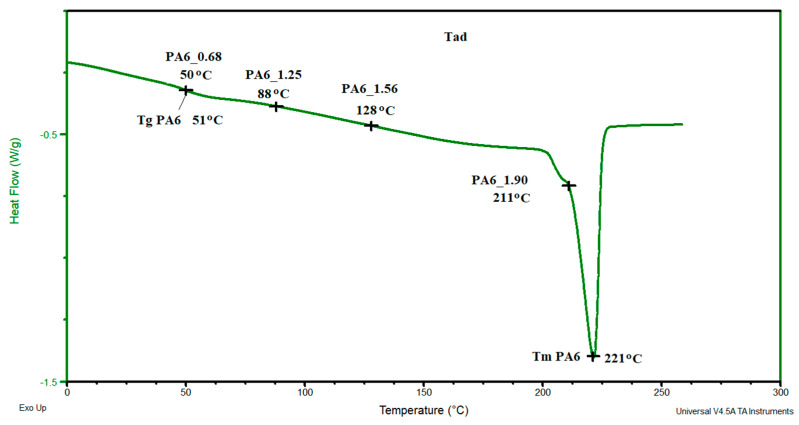
DSC thermogram of PA6 sample with the applied adiabatic temperatures that were generated during the HE process of the tested samples.

In contrast, for samples PA6_1.25 and PA6_1.56, temperatures T_m1_ and T_m2_ are, respectively, ca. 219 °C and ca. 224 °C. The first of the temperatures can be associated with the melting of phase γ and the second with phase α. For these samples, the melting points of these phases are greater than the temperatures associated with their melting. Probably, as a result of the orientation of macromolecules in the HE process, more hydrogen bonds are formed between them, which contributes to an increase in the melting points of the individual phases. The results of the crystalline phase content calculations are summarized in Table 4. As the deformation increases in the HE process, the crystalline phase proportion increases, but for the 1.90 deformation, it is slightly less than for the 1.56 deformation. To explain the reason for the differences in the phase structure of PA6 after various deformations in the HE process, the change in adiabatic temperature generated during the HE process was analyzed (Figure 14). 

In sample PA6_0.68, a temperature similar to that of PA6 was generated; in samples PA6_1.25 and PA6_1.56, respectively, temperatures of 88 and 128 °C were generated, and in PA6_1.90, a temperature of 211 °C was generated. The adiabatic temperature for sample PA6_1.90 is greater than the initial melting point of the crystalline phase PA6. The change in the adiabatic temperature generated in PA6 during the HE process is an additional reason for the changes in the structure of the samples after the HE process. A similar phenomenon of changing the structure of PA6 samples but after the process of orientation and annealing at 200° was noted by Dencheeva et al. [54].

## 4. Conclusions

The HE process on the mechanical and structural properties of polyamides has not been systematically studied so far. Most of the tests carried out on other polymers were performed under conditions of increased temperature, especially in the process of conventional extrusion.

In this study, the structure of PA6 was analyzed after a hydrostatic extrusion process performed at a linear speed of 5 mm min^−1^ and a variable deformation in the range of 0.68–1.90. As a result of the HE processes carried out at these parameters, a significant increase occurs in the strength properties determined in the static tensile test. The highest strength was obtained for samples after deformation of 1.56, and tensile strength rose by almost 500%.

As a result of microscopic observations using SEM, it was found that as a result of deformation in the HE process, the orientation of PA6 macromolecules along the deformation direction occurs.

POM images of cuttings of PA6 sample cross-sections confirmed the type of structure observed in the SEM images. As the true strain value rose, an increase was observed in the contribution of phase α in PA6 as a result of the analysis of WAXS. The results of the FTIR spectroscopic analysis confirm these observations that as the deformation increases, the proportion of the crystalline phase α rises. Thermal analysis using DSC allowed us to conclude that in the PA6 after the HE process generating deformations in the range of 0.68–1.56, the proportion of the crystalline phase α increases in PA6.

The observed changes in strength properties are due to the orientation of macromolecules and a slight increase in the proportion of the crystalline phase in these materials.

## Figures and Tables

**Table 1:** Initial mechanical properties of the PA6 polyamide.

Series	True Strain, ε	Linear Speed V (mm·s^−1^)	Extrusion Pressure p (MPa)	Adiabatic Temperature T_ad_, (°C)
PA6	0	0	0	20
PA6_0.68	0.68	5	103	50
PA6_1.25	1.25	5	180	88
PA6_1.56	1.56	5	262	128
PA6_1.90	1.90	5	430	211

**Table 2:** Description of the sample WAXS diffractograms before and after the HE process.

Series	Peak Position [deg.]			
	α_1_	α_2_	γ	Amorphous
PA6_across	20.01	23.59	21.21	20.90
PA6_0.68_across	19.85	24.07	21.06	20.51
PA6_1.25_across	19.80	-	21.09	20.08
PA6_1.56_across	-	-	20.54	20.27
PA6_1.90_across	20.26	-	20.45	20.21
PA6_along	20.09	23.65	20.56	21.25
PA6_0.68_along	19.89	23.49	21.18	21.19
PA6_1.25_along	20.16	23.24	21.85	21.40
PA6_1.56_along	20.20	23.05	21.88	21.63
PA6_1.90_along	19.95	23.33	20.99	21.61

**Table 3:** Description of component bands on WAXS diffractograms of pre-HE and post-HE of the samples.

Series	Arbitrary Units			
	α_1_	α_2_	γ	Amorphous
PA6_across	6.33	4.87	0.84	42.15
PA6_0.68_across	7.16	0.84	0.89	43.12
PA6_1.25_across	2.36	0	1.22	26.22
PA6_1.56_across	0	0	3.35	17.94
PA6_1.90_across	0.06	0	2.48	16.62
PA6_along	2.96	5.95	1.92	40.80
PA6_0.68_along	4.02	10.38	2.99	72.72
PA6_1.25_along	12.14	14.13	3.71	96.67
PA6_1.56_along	22.36	35.58	2.03	98.43
PA6_1.90_along	11.33	13.54	0.47	82.47

**Table 4:** Results of DSC analysis of PA6 samples before and after the HE process at different deformations of the samples taken from the middle part.

Series	T_g_ [°C]	T_m1_ [°C]	Peak Area—γ	T_m2_ [°C]	Peak Area—α	∆H_m_ [J/g]	XDSC [%]
PA6	50.6 ± 1.2	214 ± 2	4.2 ± 0.2	221 ± 2	4.9 ± 0.1	58.3 ± 0.9	25.3
PA6_0.68	44.2 ± 1.1	209 ± 2	5.0 ± 0.3	217 ± 3	4.5 ± 0.2	71.3 ± 2.4	31.0
PA6_1.25	53.2 ± 0.6	218 ± 1	8.8 ± 0.5	223 ± 2	3.6 ± 0.1	78.4 ± 1.9	34.1
PA6_1.56	53.2 ± 0.4	220 ± 2	10.9 ± 0.7	224 ± 2	1.3 ± 0.1	85.0 ± 2.5	37.0
PA6_1.90	53.5 ± 0.3	211 ± 1	5.2 ± 0.2	216 ± 2	6.8 ± 0.3	71.9 ± 1.7	31.3

## Data Availability

The data presented in this study are available on request from the corresponding author.
